# Urinary Neutrophil Gelatinase-Associated Lipocalin: A Useful Biomarker for Tacrolimus-Induced Acute Kidney Injury in Liver Transplant Patients

**DOI:** 10.1371/journal.pone.0110527

**Published:** 2014-10-20

**Authors:** Ayami Tsuchimoto, Haruka Shinke, Miwa Uesugi, Mio Kikuchi, Emina Hashimoto, Tomoko Sato, Yasuhiro Ogura, Koichiro Hata, Yasuhiro Fujimoto, Toshimi Kaido, Junji Kishimoto, Motoko Yanagita, Kazuo Matsubara, Shinji Uemoto, Satohiro Masuda

**Affiliations:** 1 Department of Clinical Pharmacology and Therapeutics, Kyoto University Hospital, Kyoto, Japan; 2 Department of Pharmacy, Kagawa University Hospital, Kagawa, Japan; 3 Division of Hepatobiliary-Pancreatic Surgery and Transplantation, Department of Surgery, Graduate School of Medicine, Kyoto University, Kyoto, Japan; 4 Department of Research and Development of Next Generation Medicine, Faculty of Medical Sciences, Kyushu University, Fukuoka, Japan; 5 Department of Nephrology, Graduate School of Medicine, Kyoto University, Kyoto, Japan; University Medical Center Groningen and University of Groningen, Netherlands

## Abstract

Tacrolimus is widely used as an immunosuppressant in liver transplantation, and tacrolimus-induced acute kidney injury (AKI) is a serious complication of liver transplantation. For early detection of AKI, various urinary biomarkers such as monocyte chemotactic protein-1, liver-type fatty acid-binding protein, interleukin-18, osteopontin, cystatin C, clusterin and neutrophil gelatinase-associated lipocalin (NGAL) have been identified. Here, we attempt to identify urinary biomarkers for the early detection of tacrolimus-induced AKI in liver transplant patients. Urine samples were collected from 31 patients after living-donor liver transplantation (LDLT). Twenty recipients developed tacrolimus-induced AKI. After the initiation of tacrolimus therapy, urine samples were collected on postoperative days 7, 14, and 21. In patients who experienced AKI during postoperative day 21, additional spot urine samples were collected on postoperative days 28, 35, 42, 49, and 58. The 8 healthy volunteers, whose renal and liver functions were normal, were asked to collect their blood and spot urine samples. The urinary levels of NGAL, monocyte chemotactic protein-1 and liver-type fatty acid-binding protein were significantly higher in patients with AKI than in those without, while those of interleukin-18, osteopontin, cystatin C and clusterin did not differ between the 2 groups. The area under the receiver operating characteristics curve of urinary NGAL was 0.876 (95% confidence interval, 0.800–0.951; P<0.0001), which was better than those of the other six urinary biomarkers. In addition, the urinary levels of NGAL at postoperative day 1 (p = 0.0446) and day 7 (p = 0.0006) can be a good predictive marker for tacrolimus-induced AKI within next 6 days, respectively. In conclusion, urinary NGAL is a sensitive biomarker for tacrolimus-induced AKI, and may help predict renal event caused by tacrolimus therapy in liver transplant patients.

## Introduction

Tacrolimus, a calcineurin inhibitor, is widely used as an immunosuppressant in patients undergoing liver transplantation. Although therapeutic drug monitoring helps maintain the blood concentration of tacrolimus within a narrow therapeutic range (5–15 ng/mL), preventing adverse reactions such as nephrotoxicity and neurotoxicity, adverse reactions do occur in patients with greater blood concentrations of tacrolimus [1]. One such severe adverse reaction is nephrotoxicity. Acute kidney injury (AKI) is a frequent complication of liver transplantation and its incidence has been reported to range between 36% and 78% [2]–[4]. Postoperative AKI has been reported to cause high mortality in the recipients [3], [4], and one of the main risk factors for acute renal failure after liver transplantation is calcineurin inhibitor toxicity [5], [6]. Thus, tacrolimus nephrotoxicity is a serious problem for liver transplant recipients.

Although serum creatinine (Scr) is a commonly used marker for renal function, it fails as a marker for renal injury due to the following reasons: Scr level increases after changes in glomerular filtration, and hence is thought to be a delayed marker for decreased renal function [7]. In addition, Scr is affected by non-renal factors such as age, sex, body weight, muscle mass, total body volume, and protein intake [8], [9]. Therefore, more sensitive and specific biomarkers are needed to detect AKI at an early stage.

Until now, various biomarkers for AKI have been identified, such as neutrophil gelatinase-associated lipocalin (NGAL), and liver-type fatty acid-binding protein (L-FABP). In clinical practice, NGAL serves as a good biomarker for AKI in emergency room patients [10], during septic shock [11], and after cardiac surgery [12], [13] and liver transplantation [14], [15]. L-FABP is also a good biomarker for renal damage following cisplatin-induced nephrotoxicity [16], contrast-induced nephrotoxicity [17], and septic shock induced AKI [18].

In 2007, the Acute Kidney Injury Network (AKIN) criteria for the classification and staging of AKI was published [19]. According to these criteria, an absolute increase in Scr levels of at least 0.3 mg/dL or a percentage increase of more than or equal to 50% within 48 h is defined as AKI. However, in some liver transplant recipients, the changes in Scr are gradual and cannot be evaluated according to the AKIN criteria. Therefore, new and reliable diagnostic methods for the detection of tacrolimus-induced AKI are needed. In this light, here, we attempt to identify urinary biomarkers for the early detection of tacrolimus-induced AKI in patients undergoing living-donor liver transplantation (LDLT).

## Experimental Procedures

### Patients and urine samples

A total of 21 adult patients (7 men and 14 women) who underwent LDLT at Kyoto University Hospital between August 2010 and March 2012, were enrolled in a pilot study after obtaining written informed consent. We performed power analysis using the patients who developed AKI within 14 days after liver transplantation. Among the 21 patients, 14 were diagnosed with AKI. Additionally, the patients were classified into 2 groups according to the urinary NGAL levels. The number of patients with NGAL levels lower than the cut-off value (62.0 ng/mg creatinine) was 4 among AKI-free patients and 1 among AKI patients. The power of this study was calculated as 0.606. For a power greater than 0.8, a sample size of 30 would be required.

**Figure 1:**
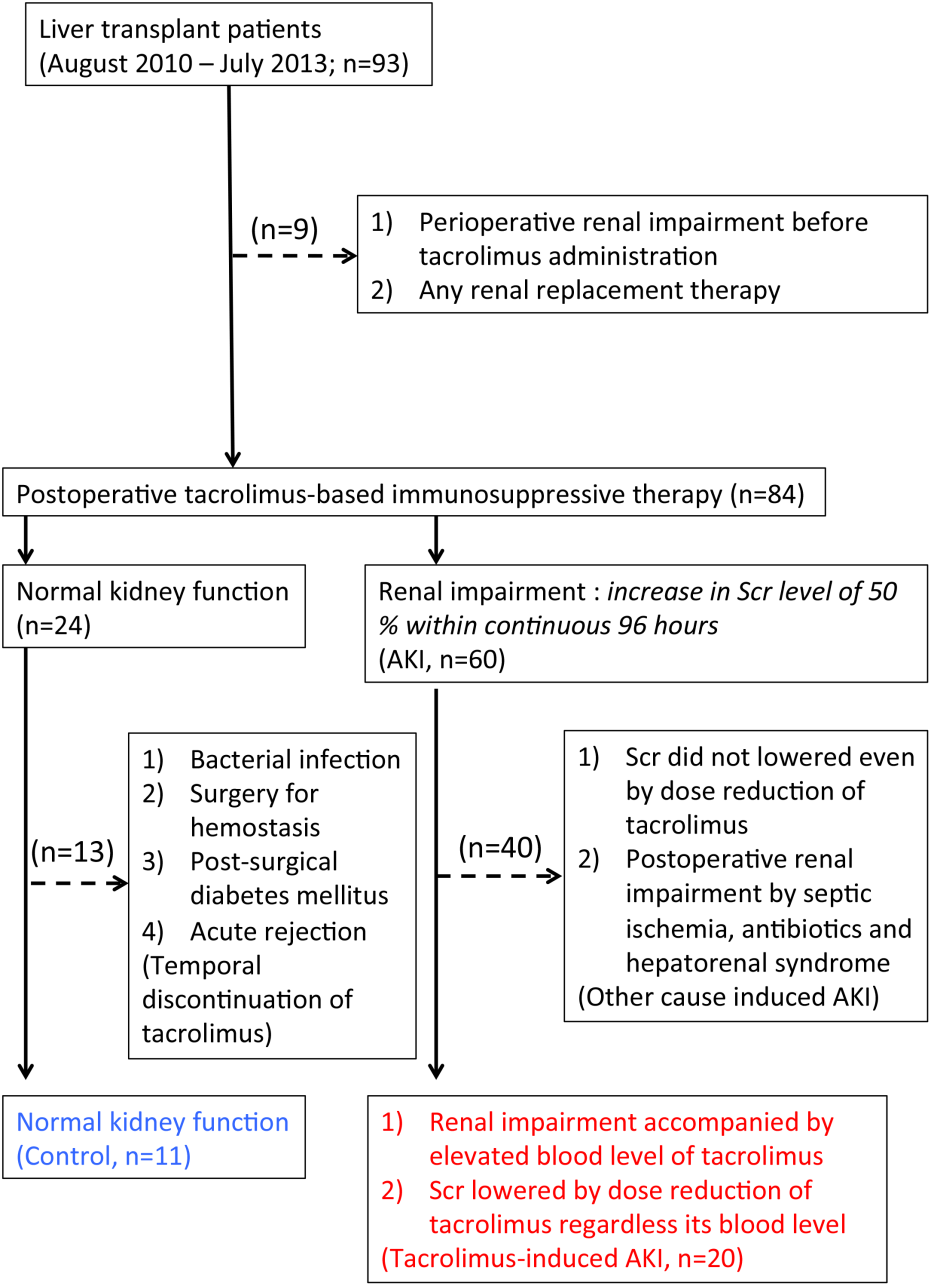
Diagnostic algorithm of tacrolimus-induced AKI in the patients after liver transplantation. Between August 2010 and July 2013, 93 patients were enrolled with the written informed consent. Nine patients with perioperative renal impairment before the administration of tacrolimus-based posttransplant immunosuppressive treatment and patients with any renal replacement therapy were excluded. Patients with renal impairment by some other causes including septic ischemia, antibiotics and hepatorenal syndrome were also excluded from this study. In addition, the patients of renal impairment with low tacrolimus levels, whose Scr levels were not changed even by the decrease of tacrolimus dosage, were also excluded indicating other causes-derived renal impairment such as tubular necrosis post-surgery. Among 24 patients with normal kidney function, 13 patients with post-transplant infectious disease, surgery for hemostasis, post-surgical diabetes mellitus and acute rejection episode were excluded for the temporal discontinuation of tacrolimus administration. Finally, the clinical data of the 11 control patients and 20 patients with tacrolimus-induced AKI were used.

Based on the results of the preliminary study, we extended the observation period to add 10 more patients. A total of 93 patients (45 men and 48 women; age, >18 years) who underwent LDLT at Kyoto University Hospital between August 2010 and July 2013, were enrolled in the present study after obtaining written informed consent. Nine patients with perioperative renal impairment before the administration of tacrolimus-based posttransplant immunosuppressive treatment, patients with renal impairment by some other causes including septic ischemia, antibiotics and hepatorenal syndrome, and patients with any renal replacement therapy were also excluded from this study. In addition, the patients of renal impairment with low tacrolimus levels, whose Scr levels were not changed even by the decrease of tacrolimus dosage, were also excluded from this study indicating other causes-derived renal impairment such as tubular necrosis post-surgery. Among them, the clinical data of the 31 liver transplant patients (12 men and 19 women) were retrospectively analyzed in the present study (Fig. 1). For comparison, 8 healthy male volunteers were also recruited with written informed consent. This study was conducted in accordance with the Declaration of Helsinki and its amendments, and was approved by the Ethics Committee of Kyoto University Graduate School and Faculty of Medicine. All patients provided written informed consent.

In all liver transplant patients, postoperative immunosuppressive therapy using tacrolimus was initiated on the morning after surgery (postoperative day 1). The blood concentration of tacrolimus was measured using a chemiluminescent enzyme immunoassay (ARCHITECT, Abbott). The daily oral dose of tacrolimus was adjusted to achieve target trough blood concentrations of 10–15 ng/mL during the first 2 weeks following surgery, approximately 10 ng/mL during the next 2 weeks, and 5–7 ng/mL thereafter [20]. Spot urine samples were collected immediately before the administration of tacrolimus on postoperative day 1 as the control urine lacking tacrolimus. After the initiation of tacrolimus therapy, urine samples were collected on postoperative days 7, 14, and 21. In patients who experienced AKI during postoperative day 21, additional spot urine samples were collected on postoperative days 28, 35, 42, 49, and 58. The 8 healthy volunteers, whose renal and liver functions were normal, were asked to collect their blood and spot urine samples. All urine samples were stored at −80°C with protease inhibitor cocktail tablets (Complete Mini, Roche Diagnostics, Mannheim, Germany).

Urinary creatinine was determined according to the Jaffé reaction by using the LabAssay Creatinine kit (Wako Pure Chemical Industries Ltd., Osaka, Japan). The biomarker candidates were measured using commercially available ELISA kits, according to the manufacturer’s instructions. NGAL, monocyte chemotactic protein-1 (MCP-1), osteopontin, and cystatin C were measured using ELISA kits purchased from R&D Systems (Minneapolis, MN). L-FABP level was determined using ELISA kits from CMIC Co., Ltd (Tokyo, Japan). Interleukin-18 (IL-18) was assessed using ELISA kits from Medical & Biological Laboratories Co. Ltd (Nagoya, Japan). Clusterin was measured using kits from AdipoGen Inc. (Incheon, Korea). The level of each urinary biomarker was normalized to urinary creatinine levels to adjust for changes in urine concentration.

### Diagnostic criteria of tacrolimus-induced AKI and data collection

Tacrolimus-induced AKI was diagnosed by the attending physicians or nephrologists, and not fully according to the AKIN criteria. They diagnosed renal impairment basically defined as an increase in Scr level of 50% within continuous 96 hours regardless the blood levels of tacrolimus was higher and/or lower than the target range. Retrospectively, the renal impairment was also diagnosed in the patients when their elevated Scr levels were lowered by the decrease of tacrolimus dosage. The AKI group comprised patients who had developed AKI, while the AKI-free group comprised patients who had not developed renal disease during the 35-day postoperative period. The clinical information, treatment process, and laboratory data of all patients were obtained from electronic medical records. The preoperative estimated glomerular filtration rate (eGFR) was calculated according to the eGFR equation for the Japanese:

eGFR = 194×Age–0.287×Scr–1.094 (×0.739, if female) [21].

### Statistical analyses

All statistical analyses were performed using Prism version 5.02 (GraphPad Software, Inc., San Diego, CA). Mann-Whitney U-test and Kruskal-Wallis test were used to compare the differences between urinary biomarker levels in AKI patients, AKI-free patients, and healthy volunteers. To compare categorical variables, we used the chi-square test or Fisher’s exact test. We determined receiver operating characteristic (ROC) curves and calculated the area under the curve, 95% confidence intervals (CI), sensitivity, specificity, positive predictive value, negative predictive value, positive likelihood ratio, and negative likelihood ratio. For ROC curve analysis, all the collected data of AKI-free group after administration of tacrolimus and those between the initiation and termination of diagnosis as renal impairment in AKI group were used. A value of P<0.05 was considered statistically significant. Probability analysis was performed according to the Kaplan-Meier method, and the outcome was compared between the subgroups by using a log-rank test. The cut-off point was examined by Youden Index [22].

## Results

### Patient characteristics

Of the 31 patients who underwent LDLT, 20 (64.5%) developed tacrolimus-induced AKI during the 35-day postoperative period. The primary diseases observed are listed in Table 1. The Child-Pugh score and model for end-stage liver disease score were significantly higher in AKI group patients than in AKI-free group patients. Because the healthy volunteers had higher muscle mass, preoperative Scr levels were significantly different between the 3 groups. Age, sex, body weight, preoperative blood urea nitrogen level, preoperative eGFR level, total dose of tacrolimus between postoperative days 1 and 21, and average blood levels of tacrolimus during the 21-day postoperative period did not differ significantly between the AKI and AKI-free groups.

**Table 1:** Patient characteristics.

	Healthy (n = 8)	AKI-free (n = 11)	AKI (n = 20)	P value
Age (years)	33.6±11.2	43.6±10.0	48.7±14.0	0.026
Sex (male/female)	8/0	4/7	8/12	
Body weight (kg)	65.1±9.8	61.0±12.4	54.7±10.2	NS
Primary disease (n)				
Biliary atresia		2	2	
Primary biliary cirrhosis		1	6	
Hepatitis C virus-related liver cancer		1	5	
Other		7	7	
ABO blood group match				
Identical		6	14	
Compatible		2	2	
Incompatible		3	4	
Child Pugh score	-	8.5±2.3	10.5±2.2	0.037
MELD score	-	15.0±6.9	18.3±5.2	NS
Donor (Living/Cadaveric), n	-	10/1	18/2	
Preoperative Scr (mg/dL)	0.78±0.06	0.61±0.19	0.69±0.24	0.024
Preoperative BUN (mg/dL)	12.6±4.8	13.6±5.8	17.1±7.0	NS
Preoperative eGFR(mL/minute/1.73 m^2^)	94.9±9.4	96.8±28.0	86.6±26.7	NS
Total dose of tacrolimus betweenPOD 1 and 21 (mg)	-	67.8±41.5	58.5±35.3	NS
Mean blood levels of tacrolimus during the 21-day postoperative period(ng/mL)	-	8.65±1.97	8.51±1.79	NS

NOTE: The results are given as mean ± standard deviation. Statistical analysis was performed using the Mann-Whitney U test and Kruskal-Wallis test.

**Abbreviations:** BUN, blood urea nitrogen; eGFR, estimated glomerular filtration rate; MELD, Model for End-stage Liver Disease; Scr, serum creatinine; POD, postoperative day.

### Diagnostic ability of urinary biomarkers

**Figure 2:**
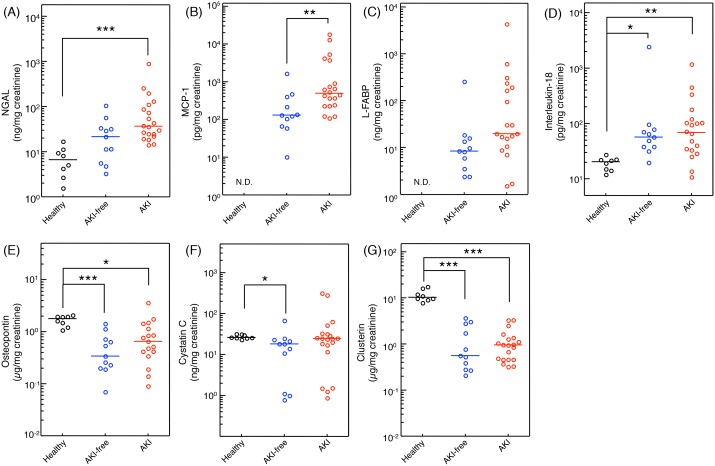
Comparison of the urinary levels of NGAL (A), MCP-1 (B), L-FABP (C), IL-18 (D), osteopontin (E), cystatin C (F), and clusterin (G) among healthy volunteers (8 measurements of 8 subjects), AKI-free group (11 measurements of 11 subjects) and AKI group (20 measurements of 20 subjects). Data were from urinary samples on postoperative day 1 immediately before the administration of tacrolimus in liver transplant patients (AKI-free group and AKI group). Data were normalized to urinary creatinine concentration and plotted on a logarithmic Y axis. Statistical analyses were performed using the Mann-Whitney U test and Kruskal-Wallis test. *<0.05, **P<0.01, ***P<0.001. NGAL, neutrophil gelatinase-associated lipocalin; MCP-1, monocyte chemotactic protein-1; L-FABP, liver-type fatty acid-binding protein; IL-18, interleukin-18, N.D., not detected.

**Figure 3:**
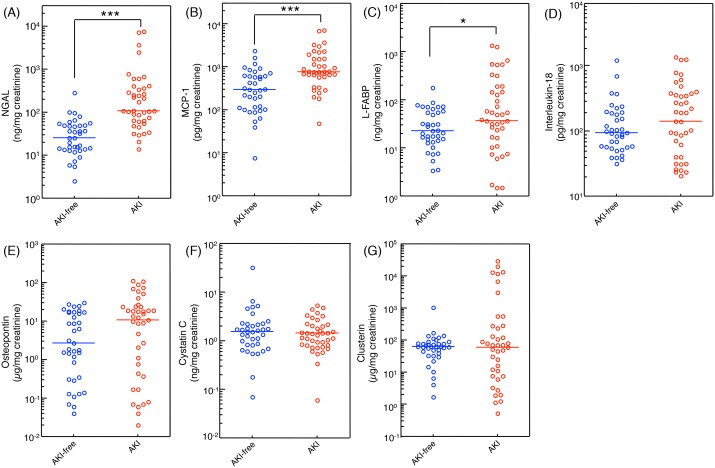
Comparison of the urinary levels of NGAL (A), MCP-1 (B), L-FABP (C), IL-18 (D), osteopontin (E), cystatin C (F), and clusterin (G) between AKI-free group (37 measurements of 11 subjects) and AKI group (40 measurements of 20 subjects). Data were from urinary samples in the post-transplant tacrolimus therapy. Data were normalized to urinary creatinine concentration and plotted on a logarithmic Y axis. Statistical analyses were performed using the Mann-Whitney U test and Kruskal-Wallis test. *P<0.05, ***P<0.001. NGAL, neutrophil gelatinase-associated lipocalin; MCP-1, monocyte chemotactic protein-1; L-FABP, liver-type fatty acid-binding protein; IL-18, interleukin-18, N.D., not detected.

**Figure 4:**
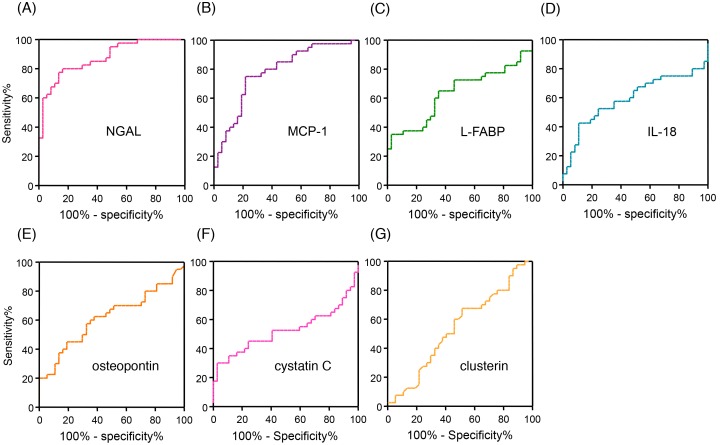
Receiver operating characteristic curve analysis of urinary NGAL (A), MCP-1 (B), L-FABP (C), IL-18 (D), osteopontin (E), cystatin C (F), and clusterin (G). Urinary biomarker levels were corrected using urinary creatinine concentrations. NGAL, neutrophil gelatinase-associated lipocalin; MCP-1, monocyte chemotactic protein-1; L-FABP, liver-type fatty acid-binding protein; IL-18, interleukin-18.

Seven urinary biomarkers were measured in the urine samples which were collected immediately before the administration of tacrolimus on postoperative day 1 of AKI and AKI-free patients, and healthy volunteers (Fig. 2). Urinary level of NGAL in the AKI group was significantly higher than that in the healthy volunteers (Fig. 2A). Basement urinary levels of IL-18 and MCP-1 were significantly higher in the patients receiving liver transplantation immediately before administration of tacrolimus on postoperative day 1 compared to healthy volunteers. Urinary levels of biomarkers during AKI (40 measurements of 20 AKI patients) and all measurements of 11 AKI-free patients (37 measurements) were summarized (Fig. 3). Urinary levels of NGAL, MCP-1 and L-FABP in AKI patients were significantly higher than those in AKI-free patients during the posttransplant course with administration of tacrolimus. However, urinary levels of IL-18, osteopontin, cystatin C, and clusterin did not differ between the AKI and AKI-free groups. To determine the specificity and sensitivity of urinary biomarkers in the diagnosis of tacrolimus-induced AKI, we performed ROC analysis (Fig. 4). The area under the curve (AUC) for ROC curve of each urinary biomarker, sensitivity, and specificity are summarized in Table 2. Based on these results, we focused on the urinary concentrations of NGAL as useful biomarker to detect tacrolimus-induced AKI in liver transplant patients.

**Table 2:** Characteristics of the urinary biomarkers.

	AUC (95% CI)	Cut-offvalue	Sensitivity(95% CI)	Specificity(95% CI)	Positivepredictive value	Negativepredictive value	Positivelikelihood ratio	Negativelikelihood ratio	P value
NGAL (ng/mgcreatinine)	0.876 (0.800–0.951)	61.0	0.78 (0.62–0.89)	0.86 (0.71–0.95)	0.83	0.74	5.57	0.26	<0.0001
MCP-1 (pg/mg creatinine)	0.781 (0.677–0.885)	642.0	0.75 (0.59–0.87)	0.78 (0.62–0.90)	0.79	0.69	3.41	0.32	<0.0001
L-FABP (ng/mg creatinine)	0.635 (0.509–0.762)	91.3	0.35 (0.21–0.52)	0.97 (0.86–1.00)	0.93	0.55	11.7	0.67	0.041
IL-18 (pg/mgcreatinine)	0.595 (0.463–0.726)	268.9	0.43 (0.27–0.59)	0.89 (0.75–0.97)	0.67	0.56	3.91	0.64	0.153
Osteopontin (µg/mg creatinine)	0.618 (0.491–0.745)	17.6	0.45 (0.29–0.62)	0.81 (0.65–0.92)	0.6	0.55	2.37	0.68	0.075
Cystatin C (ng/mg creatinine)	0.511 (0.379–0.643)	13.6	0.35 (0.21–0.51)	0.89 (0.75–0.97)	0.41	0.21	3.18	0.73	0.866
Clusterin (µg/mg creatinine)	0.521 (0.392–0.650)	1.63	0.65 (0.49–0.79)	0.49 (0.32–0.66)	0.50	0.46	1.27	0.71	0.746

**Abbreviations:** AUC, area under the curve; CI, confidence interval; IL-18, interleukin-18; L-FABP, liver-type fatty acid-binding protein; MCP-1, monocyte chemotactic protein-1; NGAL, neutrophil gelatinase-associated lipocalin.

### The changes of serum and urinary markers

**Figure 5:**
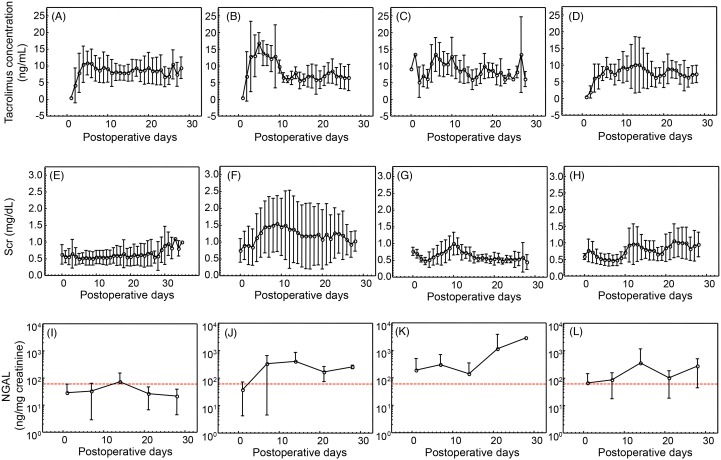
Time-dependent changes tacrolimus concentration, Scr levels and urinary NGAL concentrations. The average ± SD values of tacrolimus trough concentrations, Scr levels and urinary NGAL concentrations in the liver transplant patients who experienced AKI during the period of postoperative day 1–5 (B, F, J), during the postoperative day 6–10 (C, G, K), after the postoperative day 11 (D, H, L) and AKI-free patients (A, E, I) are summarized. The cut-off values of urinary NGAL calculated from ROC analysis were 61.0 ng/mg creatinine (red dotted line).

Next, we tried to find out the association between the concentrations of tacrolimus, Scr levels and urinary concentrations of NGAL with AKI development. In Fig. 5, the time-dependent changes of each parameter are shown. A large variation of tacrolimus concentrations and Scr was found both in AKI-free and AKI patients. Urinary concentrations of NGAL tended to be higher than the cut-off value (61.0 ng/mg creatinine) in the AKI group, but not in AKI-free group.

### Predictability of urinary NGAL

**Figure 6:**
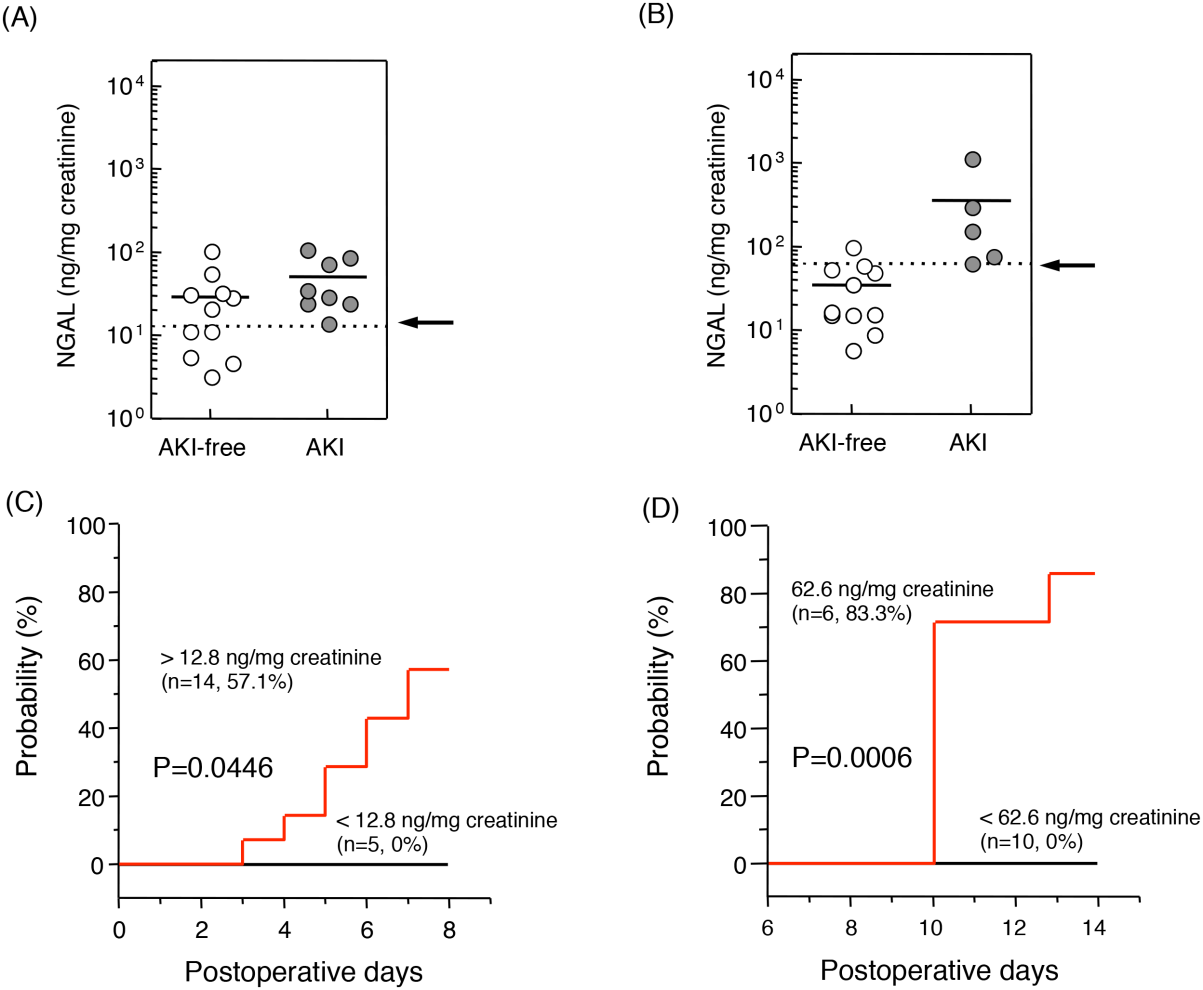
Urinary levels of NGAL in AKI and AKI-free patients. The cut-off values of urinary NGAL at postoperative day 1 (A, dotted line: 12.8 ng/mg creatinine) and postoperative day 7 (B, dotted line: 62.6 ng/mg creatinine) were evaluated using ROC curve analysis. Although the urinary level of NGAL in the AKI group was similar to that of the AKI-free group at postoperative day 1 (**A**), that at postoperative day 7 was markedly higher in the AKI group than in the AKI-free group (**B**). The probability of AKI developing between postoperative days 1 and 7 (**C**) and between postoperative days 8 and 14 (**D**) was examined using Kaplan-Meier analysis and a log-rank test. Statistical analysis was performed using the Mann-Whitney U test. **P<0.01. NGAL, neutrophil gelatinase-associated lipocalin.

Because the urinary level of NGAL was found to have the highest sensitivity and specificity in detecting tacrolimus-induced AKI in liver transplant patients, we examined whether the urinary level of NGAL could predict the occurrence of tacrolimus-induced AKI in patients after LDLT. The 20 patients who developed AKI during the 35 days after surgery were categorized into the 3 groups based on the time of diagnosis of tacrolimus-induced AKI: 8 patients developed tacrolimus-induced AKI within 7 postoperative days (AKI 1–7), 5 developed it between postoperative days 8 and 14 (AKI 8–14), and the remaining 7 developed it after postoperative day 15. The relationship between urinary level of NGAL at postoperative day 1 and the development of AKI in next 6 days was assessed. Although no statistically significant difference was found in the urinary NGAL levels at postoperative day 1 between the AKI 1–7 and AKI-free groups (Fig. 6A), the urinary NGAL levels at postoperative day 7 of the AKI 8–14 group was markedly higher than that of the AKI-free group (Fig. 6B). After dividing the samples by using the threshold values by ROC curves, the probability of tacrolimus-induced AKI was examined based on the urinary NGAL levels before AKI development, according to the Kaplan-Meier method. As shown in Figs. 6C and 6D, high urinary levels of NGAL at postoperative day 1 and 7, respectively, were correlated with the probability of AKI.

## Discussion

In this study, we examined various candidate urinary biomarkers for the early detection and/or prediction of tacrolimus-induced AKI in patients who had received LDLT. Thus far, similar studies were conducted in patients with ischemic AKI that developed after cardiovascular surgery and or in patients with severe infectious AKI [8], [23], [24]. Recently, on the basis of microarray analysis with isolated renal proximal tubules, we found that urinary levels of MCP-1 could serve as sensitive and specific biomarkers for cisplatin-induced nephrotoxicity in rats [25]. Cisplatin-induced renal toxicity has been found to initiate at the proximal straight tubules, gradually transducing into glomerular damage, tubular apoptosis, and interstitial damage [26]. However, the molecular mechanisms underlying tacrolimus-induced nephrotoxicity remain unclear, although they are considered different from that of cisplatin [27], [28]. In the present study, urinary level of NGAL was found to be a useful biomarker for tacrolimus-induced nephrotoxicity in LDLT patients.

In patients with end-stage liver disease, many complications in addition to hepatic dysfunction have been reported, such as renal impairment due to hepatorenal syndrome, respiratory failure due to hepatopulmonary syndrome, coagulation disorder, edema, and consciousness disorder due to hepatic coma. In addition, the surgical procedure of LDLT is highly invasive with respect to renal function. McCauley et al. [29] reported that the peak level of Scr, which was higher than 3 mg/dL, carried a significant risk of death in liver transplant patients. Fraley et al. [30] showed that the mortality of patients with post-liver transplant AKI was 41%, whereas that of patients without post-liver transplant AKI was 5%. In the present study, the urinary levels of MCP-1 and L-FABP in AKI-free patients were markedly higher than those of healthy subjects. However, the urinary L-FABP levels between AKI-free patients and patients of AKI group were not significantly different. Among 7 biomarker candidates, only urinary level of NGAL in AKI-free patients was similar with that of healthy subjects and significantly lower than those of AKI group, suggesting that urinary NGAL level rapidly decreased in the control prior to the administration of tacrolimus by the morning of postoperative day 1. Taken together, urinary NGAL would be sensitive biomarkers for the detection of tacrolimus-induced AKI in patients after LDLT. Because power analysis showed that the r-value of 0.369 in the present study was relatively moderate in the examination of urinary NGAL, further analysis in future with larger sample size should be examined to find the accuracy of the present results.

NGAL, a 25-kDa protein, was purified from human neutrophils [31], and is expressed at very low concentrations in the bone marrow and several human tissues, such as those of the trachea, kidney, lung, and stomach [32]. NGAL is one of the most upregulated genes and overexpressed proteins after renal ischemia, and urinary levels of NGAL increase soon after ischemic renal injury in mouse and rat models [33]. The Ngal: siderophore: Fe complex upregulates heme oxygenase-1 to preserve proximal tubules and prevent cell death [34]. NGAL has been reported to be a useful marker for renal ischemic injury such as that occurring after cardiac surgery [12], [13] and liver transplantation [14], [15], and for acute tubular injury such as cisplatin-induced AKI [35] and contrast-induced nephropathy [36]. Calcineurin inhibitor causes structural damage to the straight segment of the proximal tubule [37] and renal vasoconstriction, which is mediated by the renal sympathetic nervous system [38]. Thus, these findings suggest that NGAL is upregulated and detected in the urine of patients with tacrolimus-induced vasoconstriction and structural renal damage.

Urinary levels of NGAL at postoperative day 7 in the AKI 8–14 group were significantly higher than those in the AKI-free group, indicating that the urinary levels of NGAL at postoperative day 7 can be a good predictive marker for tacrolimus-induced AKI. Wagener et al. [15] reported that urinary level of NGAL/urine creatinine ratio could predict postoperative AKI between 3 and 18 h after liver transplantation [15]. At postoperative day 1, urinary levels of NGAL may reflect renal injury caused by the liver transplant operation. However, of the 7 urinary biomarkers examined, NGAL, osteopontin, and clusterin are synthesized in the proximal as well as distal tubules [8]. On the other hand, MCP-1, L-FABP, and IL-18 are specifically synthesized in the proximal tubules. In a histological examination, Morgan et al. [39] reported that tacrolimus-induced nephrotoxicity caused interstitial fibrosis. In addition, excess expression of transforming growth factor beta 1 has been shown to be related to the interstitial fibrosis caused by tacrolimus-induced nephrotoxicity [40], [41]. These findings suggest that the origin of urinary NGAL might be the proximal as well as distal tubules. Therefore, a biomarker synthesized at both the proximal and distal tubules such NGAL could associate well with renal vasoconstriction and interstitial fibrosis caused by tacrolimus-induced nephrotoxicity.

In conclusion, the urinary level of NGAL can serve as a sensitive and predictive biomarker for tacrolimus-induced AKI, and urinary NGAL-based monitoring of renal functions in liver transplant recipients may be a convenient and effective way of managing tacrolimus-induced AKI. However, further studies on larger populations of patients, healthy volunteers, and/or other organ transplant patients are required.

## References

[1] Masuda S, Inui K (2006) An up-date review on individualized dosage adjustment of calcineurin inhibitors in organ transplant patients. Pharmacol Ther 112: 184–198. 16759707 10.1016/j.pharmthera.2006.04.006

[2] Barri YM, Sanchez EQ, Jennings LW, Melton LB, Hays S, et al (2009) Acute Kidney Injury Following Liver Transplantation: Definition and Outcome. Liver Transplantation 15: 475–483. 19399734 10.1002/lt.21682

[3] Lima EQ, Zanetta DMT, Castro I, Massarollo PCB, Mies S, et al (2003) Risk factors for development of acute renal failure after liver transplantation. Renal Failure 25: 553–560. 12911159 10.1081/jdi-120022546

[4] O'Riordan A, Wong V, McQuillan R, McCormick PA, Hegarty JE, et al (2007) Acute renal disease, as defined by the RIFLE criteria, post-liver transplantation. American Journal of Transplantation 7: 168–176. 17109735 10.1111/j.1600-6143.2006.01602.x

[5] Cabezuelo JB, Ramirez P, Rios A, Acosta F, Torres D, et al (2006) Risk factors of acute renal failure after liver transplantation. Kidney International 69: 1073–1080. 16528257 10.1038/sj.ki.5000216

[6] McCauley J, Vanthiel DH, Starzl TE, Puschett JB (1990) Acute and Chronic-Renal-Failure in Liver-Transplantation. Nephron 55: 121–128. 2362625 10.1159/000185938 PMC2957102

[7] Vaidya VS, Ferguson MA, Bonventre JV (2008) Biomarkers of acute kidney injury. Annual Review of Pharmacology and Toxicology 48: 463–493. 10.1146/annurev.pharmtox.48.113006.094615 PMC2742480 17937594

[8] Bonventre JV, Vaidya VS, Schmouder R, Feig P, Dieterle F (2010) Next-generation biomarkers for detecting kidney toxicity. Nature Biotechnology 28: 436–440. 10.1038/nbt0510-436 PMC3033582 20458311

[9] Chariton MR, Wall WJ, Ojo AO, Gines P, Textor S, et al (2009) Report of the First International Liver Transplantation Society Expert Panel Consensus Conference on Renal Insufficiency in Liver Transplantation. Liver Transplantation 15: S1–S34. 10.1002/lt.21877 19877213

[10] Nickolas TL, O'Rourke MJ, Yang J, Sise ME, Canetta PA, et al (2008) Sensitivity and specificity of a single emergency department measurement of urinary neutrophil gelatinase-associated lipocalin for diagnosing acute kidney injury. Annals of Internal Medicine 148: 810–U821. 18519927 10.7326/0003-4819-148-11-200806030-00003 PMC2909852

[11] Wheeler DS, Devarajan P, Ma D, Harmon K, Monaco M, et al (2008) Serum neutrophil gelatinase-associated lipocalin (NGAL) as a marker of acute kidney injury in critically ill children with septic shock. Critical Care Medicine 36: 1297–1303. 18379258 10.1097/CCM.0b013e318169245a PMC2757115

[12] Bennett M, Dent CL, Ma Q, Dastrala S, Grenier F, et al (2008) Urine NGAL predicts severity of acute kidney injury after cardiac surgery: A prospective study. Clinical Journal of the American Society of Nephrology 3: 665–673. 18337554 10.2215/CJN.04010907 PMC2386703

[13] Mishra J, Dent C, Tarabishi R, Mitsnefes MM, Ma Q, et al (2005) Neutrophil gelatinase-associated lipocalin (NGAL) as a biomarker for acute renal injury after cardiac surgery. Lancet 365: 1231–1238. 15811456 10.1016/S0140-6736(05)74811-X

[14] Niemann CU, Walia A, Waldman J, Davio M, Roberts JP, et al (2009) Acute Kidney Injury During Liver Transplantation as Determined by Neutrophil Gelatinase-Associated Lipocalin. Liver Transplantation 15: 1852–1860. 19938135 10.1002/lt.21938

[15] Wagener G, Minhaz M, Mattis FA, Kim M, Emond JC, et al (2011) Urinary neutrophil gelatinase-associated lipocalin as a marker of acute kidney injury after orthotopic liver transplantation. Nephrology Dialysis Transplantation 26: 1717–1723. 10.1093/ndt/gfq770 PMC3145384 21257679

[16] Negishi K, Noiri E, Sugaya T, Li S, Megyesi J, et al (2007) A role of liver fatty acid-binding protein in cisplatin-induced acute renal failure. Kidney Int 72: 348–358. 17495861 10.1038/sj.ki.5002304

[17] Manabe K, Kamihata H, Motohiro M, Senoo T, Yoshida S, et al (2012) Urinary liver-type fatty acid-binding protein level as a predictive biomarker of contrast-induced acute kidney injury. Eur J Clin Invest 42: 557–563. 22070248 10.1111/j.1365-2362.2011.02620.x

[18] Nakamura T, Sugaya T, Koide H (2009) Urinary liver-type fatty acid-binding protein in septic shock: effect of polymyxin B-immobilized fiber hemoperfusion. Shock 31: 454–459. 18838948 10.1097/SHK.0b013e3181891131

[19] Mehta RL, Kellum JA, Shah SV, Molitoris BA, Ronco C, et al.. (2007) Acute Kidney Injury Network: report of an initiative to improve outcomes in acute kidney injury. Critical Care 11. 10.1186/cc5713 PMC2206446 17331245

[20] Uesugi M, Kikuchi M, Shinke H, Omura T, Yonezawa A, et al (2014) Impact of cytochrome P450 3A5 polymorphism in graft livers on the frequency of acute cellular rejection in living-donor liver transplantation. Pharmacogenet Genomics 24: 356–366. 24911663 10.1097/FPC.0000000000000060

[21] Matsuo S, Imai E, Horio M, Yasuda Y, Tomita K, et al (2009) Revised Equations for Estimated GFR From Serum Creatinine in Japan. American Journal of Kidney Diseases 53: 982–992. 19339088 10.1053/j.ajkd.2008.12.034

[22] Fluss R, Faraggi D, Reiser B (2005) Estimation of the Youden Index and its associated cutoff point. Biom J 47: 458–472. 16161804 10.1002/bimj.200410135

[23] Devarajan P (2011) Biomarkers for the early detection of acute kidney injury. Curr Opin Pediatr 23: 194–200. 21252674 10.1097/MOP.0b013e328343f4dd PMC3257513

[24] Ferguson MA, Vaidya VS, Bonventre JV (2008) Biomarkers of nephrotoxic acute kidney injury. Toxicology 245: 182–193. 18294749 10.1016/j.tox.2007.12.024 PMC4038970

[25] Nishihara K, Masuda S, Shinke H, Ozawa A, Ichimura T, et al.. (2013) Urinary chemokine (C-C motif) ligand 2 (monocyte chemotactic protein-1) as a tubular injury marker for early detection of cisplatin-induced nephrotoxicity. Biochem Pharmacol in press. 10.1016/j.bcp.2012.12.019 PMC4077184 23291264

[26] Pabla N, Dong Z (2008) Cisplatin nephrotoxicity: mechanisms and renoprotective strategies. Kidney Int 73: 994–1007. 18272962 10.1038/sj.ki.5002786

[27] Peralta CA, Katz R, Bonventre JV, Sabbisetti V, Siscovick D, et al (2012) Associations of Urinary Levels of Kidney Injury Molecule 1 (KIM-1) and Neutrophil Gelatinase-Associated Lipocalin (NGAL) With Kidney Function Decline in the Multi-Ethnic Study of Atherosclerosis (MESA). American Journal of Kidney Diseases 60: 904–911. 22749388 10.1053/j.ajkd.2012.05.014 PMC3690926

[28] Gijsen VM, Madadi P, Dube MP, Hesselink DA, Koren G, et al (2012) Tacrolimus-induced nephrotoxicity and genetic variability: a review. Ann Transplant 17: 111–121. 10.12659/aot.883229 22743729

[29] McCauley J, Van Thiel DH, Starzl TE, Puschett JB (1990) Acute and chronic renal failure in liver transplantation. Nephron 55: 121–128. 2362625 10.1159/000185938 PMC2957102

[30] Fraley DS, Burr R, Bernardini J, Angus D, Kramer DJ, et al (1998) Impact of acute renal failure on mortality in end-stage liver disease with or without transplantation. Kidney International 54: 518–524. 9690218 10.1046/j.1523-1755.1998.00004.x

[31] Kjeldsen L, Johnsen AH, Sengelov H, Borregaard N (1993) Isolation and Primary Structure of Ngal, A Novel Protein Associated with Human Neutrophil Gelatinase. Journal of Biological Chemistry 268: 10425–10432. 7683678

[32] Cowland JB, Borregaard N (1997) Molecular characterization and pattern of tissue expression of the gene for neutrophil gelatinase-associated lipocalin from humans. Genomics 45: 17–23. 9339356 10.1006/geno.1997.4896

[33] Mishra J, Ma Q, Prada A, Mitsnefes M, Zahedi K, et al (2003) Identification of neutrophil gelatinase-associated lipocalin as a novel early urinary biomarker for ischemic renal injury. Journal of the American Society of Nephrology 14: 2534–2543. 14514731 10.1097/01.asn.0000088027.54400.c6

[34] Mori K, Lee HT, Rapoport D, Drexler IR, Foster K, et al (2005) Endocytic delivery of lipoccalin-siderophore-iron complex rescues the kidney from ischemia-reperfusion injury. Journal of Clinical Investigation 115: 610–621. 15711640 10.1172/JCI23056 PMC548316

[35] Gaspari F, Cravedi P, Mandala M, Perico N, de Leon FR, et al (2010) Predicting Cisplatin-Induced Acute Kidney Injury by Urinary Neutrophil Gelatinase-Associated Lipocalin Excretion: A Pilot Prospective Case-Control Study. Nephron Clinical Practice 115: C154–C160. 20407275 10.1159/000312879

[36] Hirsch R, Dent C, Pfriem H, Allen J, Beekman RH III, et al.. (2007) NGAL is an early predictive biomarker of contrast-induced nephropathy in children. Pediatric Nephrology 22. 10.1007/s00467-007-0601-4 17874137

[37] Whiting PH, Thomson AW, Blair JT, Simpson JG (1982) Experimental Cyclosporin a Nephrotoxicity. British Journal of Experimental Pathology 63: 88–94. 7066186 PMC2040750

[38] Murray BM, Paller MS, Ferris TF (1985) Effect of Cyclosporine Administration on Renal Hemodynamics in Conscious RATS. Kidney International 28: 767–774. 3910916 10.1038/ki.1985.196

[39] Morgan C, Sis B, Pinsk M, Yiu V (2011) Renal interstitial fibrosis in children treated with FK506 for nephrotic syndrome. Nephrol Dial Transplant 26: 2860–2865. 21303962 10.1093/ndt/gfq813

[40] Ogutmen B, Tuglular S, Cakalagaoglu F, Ozener C, Akoglu E (2006) Transforming growth factor-beta1, vascular endothelial growth factor, and bone morphogenic protein-7 expression in tacrolimus-induced nephrotoxicity in rats. Transplant Proc 38: 487–489. 16549155 10.1016/j.transproceed.2005.12.048

[41] Shihab FS, Bennett WM, Tanner AM, Andoh TF (1997) Mechanism of fibrosis in experimental tacrolimus nephrotoxicity. Transplantation 64: 1829–1837. 9422427 10.1097/00007890-199712270-00034

